# Lipid Trafficking at Membrane Contact Sites During Plant Development and Stress Response

**DOI:** 10.3389/fpls.2019.00002

**Published:** 2019-01-15

**Authors:** Morgane Michaud, Juliette Jouhet

**Affiliations:** UMR 5168 CNRS, CEA, INRA, Laboratoire de Physiologie Cellulaire et Végétale, Université Grenoble Alpes, Grenoble, France

**Keywords:** lipid transfer, membrane contact sites, higher plants, stress, membrane homeostasis

## Abstract

The biogenesis of cellular membranes involves an important traffic of lipids from their site of synthesis to their final destination. Lipid transfer can be mediated by vesicular or non-vesicular pathways. The non-vesicular pathway requires the close apposition of two membranes to form a functional platform, called membrane contact sites (MCSs), where lipids are exchanged. These last decades, MCSs have been observed between virtually all organelles and a role in lipid transfer has been demonstrated for some of them. In plants, the lipid composition of membranes is highly dynamic and can be drastically modified in response to environmental changes. This highlights the importance of understanding the mechanisms involved in the regulation of membrane lipid homeostasis in plants. This review summarizes our current knowledge about the non-vesicular transport of lipids at MCSs in plants and its regulation during stress.

## Introduction

**FIGURE 1:**
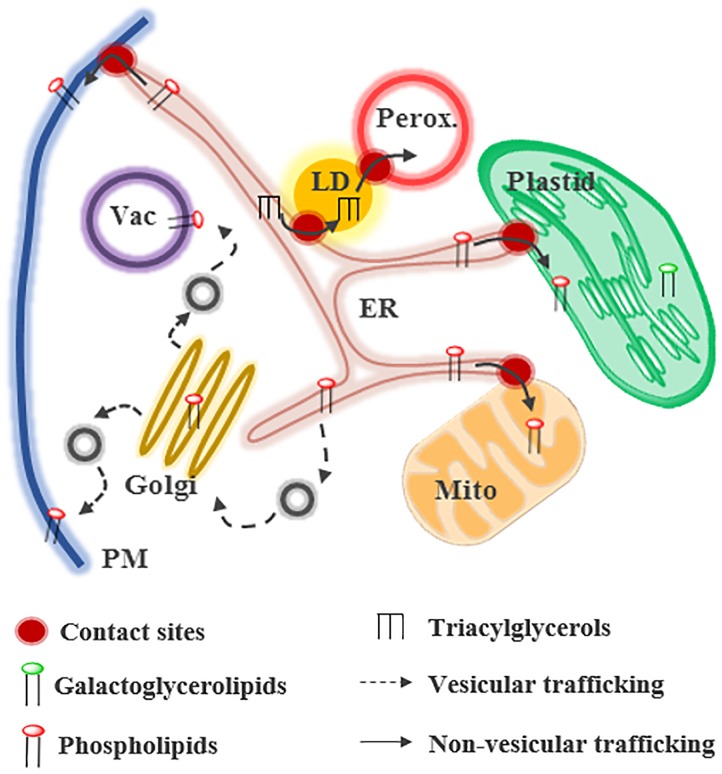
Glycerolipid trafficking in plant cells. Lipids can be transferred by vesicular or non-vesicular routes at contact sites between membranes. Phospholipids and triacylglycerols are mainly synthesized in the endoplasmic reticulum (ER) whereas galactoglycerolipids are synthesized in plastids. In presence of phosphate (Pi), galactoglycerolipids are located primary in plastids and extra-plastidial membranes contain phospholipids. LD, lipid droplet; Mito, mitochondria; Perox, peroxisome.

Cellular membranes are composed of a specific assembly of lipids and proteins defining their functions and identities. Different types of lipids are found in membranes, the most abundant being glycerolipids present in all membranes, then sterols, and sphingolipids particularly enriched in the PM (Li-Beisson et al., 2013). Glycerolipids are composed of a glycerol backbone esterified with two FAs and of a polar head, which composition defines different families, such as the phospholipids and the galactoglycerolipids. A third FA can also be esterified on the glycerol backbone to form the TAGs, which are reserve lipids stored in LDs. In plants, FAs are synthesized in plastids and a fraction is exported to fulfill the synthesis of glycerolipids in extra-plastidial membranes (Li-Beisson et al., 2013, 2017). Phospholipids contain a Pi group in their polar head. They are mainly synthesized in the ER from PA and constitute the major glycerolipids present within extra-plastidial membranes (Figure 1; Li-Beisson et al., 2013; Michaud et al., 2017). Galactoglycerolipids do not contain a Pi group but one or two galactose residues to form the MGDG and DGDG, respectively. These lipids are synthesized in plastids and are mainly located in this compartment under normal growth conditions (Boudiere et al., 2012). The biosynthesis of galactoglycerolipids inside chloroplasts relies on precursor lipids synthesized in the ER, by the so-called eukaryotic pathway, and in some plant species, from precursors synthesized directly in plastids by the prokaryotic pathway (Mongrand et al., 1998; Block and Jouhet, 2015; Mueller-Schuessele and Michaud, 2018). Under certain stresses, like Pi starvation, DGDG is also found in extra-plastidial membranes (Hartel et al., 2000; Dormann and Benning, 2002; LaBrant et al., 2018). Thus, because lipids are synthesized at specific locations in the cell and have then to be distributed to other cell compartments, an important and complex traffic of lipids between membranes is required to sustain organelle biogenesis in cells.

Lipid transfer between organelles can be mediated by vesicular or non-vesicular pathways. Some compartments, such as the Golgi and the PM, import lipids *via* these two pathways (Jurgens, 2004). Some other organelles, like mitochondria or plastids, rely mostly on non-vesicular routes for the biogenesis of their membranes (Figure 1; (Hurlock et al., 2014; Block and Jouhet, 2015; Michaud et al., 2017). Non-vesicular lipid transfer requires, (1) the desorption of the lipid from the donor membrane, which is the limiting step of the process, (2) the movement of this hydrophobic molecule across a soluble phase, and (3) its insertion into the acceptor membrane. The spontaneous transfer of glycerolipids is a very long process that has been judged irrelevant *in vivo* (Phillips et al., 1987). Some proteins, called LTPs, facilitate non-vesicular lipid transfer. LTPs are composed of a hydrophobic pocket accommodating lipids and stimulate the steps 1 and 2 of the transfer process (Wong et al., 2017). Lipid exchange is thought to occur at MCSs between organelles. MCSs are regions where two membranes are at a distance of less than 30 nm and form functional platforms (Phillips and Voeltz, 2016; Wu et al., 2018). They are formed and maintained by tethering proteins, which bridge the two membranes. Even if MCSs have been observed between almost all organelles in many species (Perez-Sancho et al., 2016; Cohen et al., 2018), their composition and the mechanisms involved in lipid transfer are still largely unknown, particularly in plants. This review summarizes our current knowledge about glycerolipid transfer at MCSs in plants and the regulation of this process in response to stress.

## Lipid Trafficking During Plant Development

### Lipid Trafficking at ER-Plastids MCSs

Lipid transport from ER to plastids is thought to occur by non-vesicular transfer at MCSs (Hurlock et al., 2014; Block and Jouhet, 2015; LaBrant et al., 2018; Mueller-Schuessele and Michaud, 2018). ER-plastids MCSs have been observed by microscopy and a fraction of the ER is tightly attached to purified chloroplasts (Renaudin and Capdepon, 1977; Andersson et al., 2007; Schattat et al., 2011). This fraction, called PLAM, has a particular protein and lipid composition and contains a PC synthase activity, supporting a role of ER-plastids MCSs in lipid metabolism (Andersson et al., 2007). Plastids are able to form protrusions of around 1 μm of diameter called stromules. Stromules are highly dynamic structures that are regulated during plant development or in response to different biotic or abiotic stresses (Kohler and Hanson, 2000; Hanson and Sattarzadeh, 2011; Krenz et al., 2014). They are able to interact with other cell compartments, including the ER (Schattat et al., 2011). However, the role of stromule-organelle interactions in lipid exchanges is still unknown and remains to be investigated.

It is well known that PC synthesized in the ER is the main precursor for galactoglycerolipids synthesis in plastids (Andersson et al., 2004; Benning, 2008). MGDG synthesis is mainly catalyzed by the MGDG synthase 1 (MGD1), located in the inner envelope (IE) of plastids. Then, DGD synthases (DGD) located in the plastid OE synthesize DGDG from MGDG. MGD1 uses diacylglycerol (DAG) as a substrate, meaning that DAG, generated from PC synthesized in the ER, has to be located in the IE. It is not clear where the DAG required for this process is initially generated and there are still debates concerning which lipid(s) are translocated from the ER to plastids. Currently, PC, lyso-PC (i.e., a PC with one FA), PA and/or DAG have been proposed to be the transported molecules. It is likely that several mechanisms involved in the transport of different lipids co-exist in cells to ensure the biogenesis of plastid membranes in different conditions.

**FIGURE 2:**
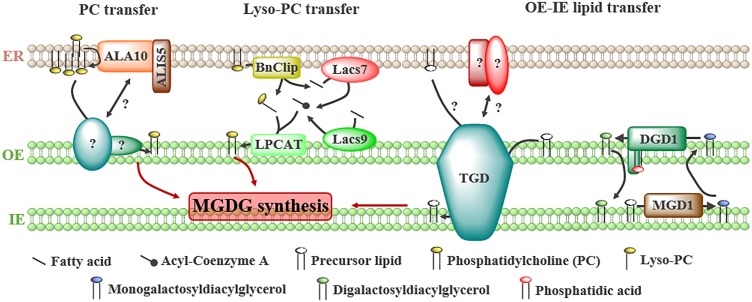
Proteins involved in the transport of lipids from the ER to plastids and between the outer (OE) and the inner (IE) envelope of plastids to fulfill galactoglycerolipid synthesis. Several precursors have been proposed to be transported from ER: (1) phosphatidylcholine (PC), whose transfer is stimulated by the flippase ALA10 located at ER-plastids junctions by interacting with ALIS5, (2) lyso-PC, generated from PC by a lipase, BnClip1, and reacylated in the OE by a lyso-PC acyltransferase (LPCAT) from acyl-Coenzyme A synthesized at ER-plastids contact sites by the long-chain acyl-CoA synthases LACS7 and LACS9, (3) unknown precursors or galactolipids transported from the OE to the inner envelope (IE) by the trigalactosyldiacylglycerol (TGD) complex or the digalactosyldiacylglycerol (DGDG) synthase DGD1.

Isolated plastids are able to uptake PC, but not PE, coming from microsomes or liposomes (i.e., small vesicles mimicking membranes), supporting the transfer of PC molecules from the ER to plastids (Andersson et al., 2004; Yin et al., 2015). In addition, a lipid flippase, ALA10, able to translocate phospholipids from one leaflet of a membrane to another, was reported to stimulate PC export from the ER in *Arabidopsis thaliana* (Poulsen et al., 2015; Botella et al., 2016). When ALA10 interacts with ALIS5, the protein is located in the ER in close proximity to plastids (Botella et al., 2016; Figure 2). ALA10 stimulates leaf growth and increases the ratio of MGDG/PC, suggesting that MGDG synthesis from PC *via* the eukaryotic pathway is stimulated by this protein. The authors proposed that ALA10 might create a specific lipid environment in the ER favoring lipid transfer by flipping specific PC species from the luminal to the cytosolic leaflet of the ER (Botella et al., 2016). Once in the plastid envelope, PC might be degraded by phospholipases to form PA, which activates MGD1 and promotes galactoglycerolipids and plastid membranes biogenesis (Dubots et al., 2010; Botella et al., 2016).

The transport of lyso-PC from the ER to plastids seems an advantageous model as lyso-PC molecules are soluble enough to freely diffuse through the cytosol (Somerharju, 2015). This transfer is supported by pulse-chase experiments following the import of ER-synthesized lipids in leek plastids (Mongrand et al., 2000). In addition, the presence of enzymes required to generate lyso-PC in the ER and to synthesize PC by acylation after transfer in the OE of plastids have been demonstrated (Figure 2). Indeed, a lipase (BnClip1), involved in plastid development, is located at ER-plastids MCSs in *Brassica napus* (Tan et al., 2011) and might generate lyso-PC. Two long-chain acyl-CoA synthases, LACS7 and LACS9 located in the ER and plastids, respectively, were shown to influence the eukaryotic pathway of plastid lipids synthesis in *A. thaliana* (Jessen et al., 2015). It was proposed that these enzymes synthesize a specific pool of acyl-CoA at ER-plastids MCSs to sustain galactoglycerolipids synthesis (Figure 2; Jessen et al., 2015). Finally, a lyso-PC acyltransferase (LPCAT) activity was detected in leek plastid envelope (Bessoule et al., 1995).

The transfer of PA to plastids was suggested because two subunits of a complex transporting lipid precursors *via* the eukaryotic pathway, the TGD (Trigalactosyldiacylglycerol) complex, are able to bind PA (Awai et al., 2006; Wang et al., 2012). The composition and role of the TGD complex in plastid membranes biogenesis have been recently reviewed (Hurlock et al., 2014; LaBrant et al., 2018; Mueller-Schuessele and Michaud, 2018). The TGD complex is located at the interface between the OE and the IE (Figure 2) and is composed of five subunits, three of them (TGD1, 2, and 3) forming an ABC transporter in the IE, TGD4 being in the OE and TGD5 bridging the OE subunit TGD4 with the TGD1, 2, 3 complex in the IE. This complex transfers lipids from the OE to the IE to feed the MGD1 enzyme with precursors coming from the eukaryotic pathway. Whether it also transfers lipids from the ER to the OE and the nature of the transported lipid(s) are still unknown. TGD2 and TGD4 are able to bind PA (Awai et al., 2006; Wang et al., 2012), but this binding might reflect a regulatory role of PA, like for MGD1, rather than a PA transport activity. Recently, it has been suggested that DGD1 could also be involved in the transfer of lipids between the OE and the IE of plastids (Kelly et al., 2016). The N-terminal extension (NDGD1) of DGD1, required for DGD1 insertion in the OE, is essential for the synthesis of DGDG in the OE (Figure 2; Kelly et al., 2016). *In vitro*, purified NDGD1 is able to bind PA and triggers liposome fusion. It was proposed that NDGD1 mediates fusion or hemi-fusion of plastid envelopes by creating a PA-enriched environment, favoring the transport of galactoglycerolipids and/or precursors between both membranes (Kelly et al., 2016). TGD2 is also able to destabilize liposomes *in vitro* (Roston et al., 2011), thus it would be of interest to investigate whether the TGD complex and the N-terminal extension of DGD1 act in concert or not to modulate membrane structures and to favor lipid transfer between OE and IE.

The role of DAG in the regulation of galactoglycerolipids synthesis is supported by a mathematical modeling of the lipid fluxes required between OE and IE to sustain membrane biogenesis (Marechal and Bastien, 2014). This model shows that, if PA was the main imported molecule, a huge, non-physiologically relevant concentration of PA in the IE would be required to sustain plastid lipids synthesis (Marechal and Bastien, 2014). Thus, this model favors the import of DAG precursor and a role of PA in the regulation of the galactoglycerolipid synthesis pathways.

**FIGURE 3:**
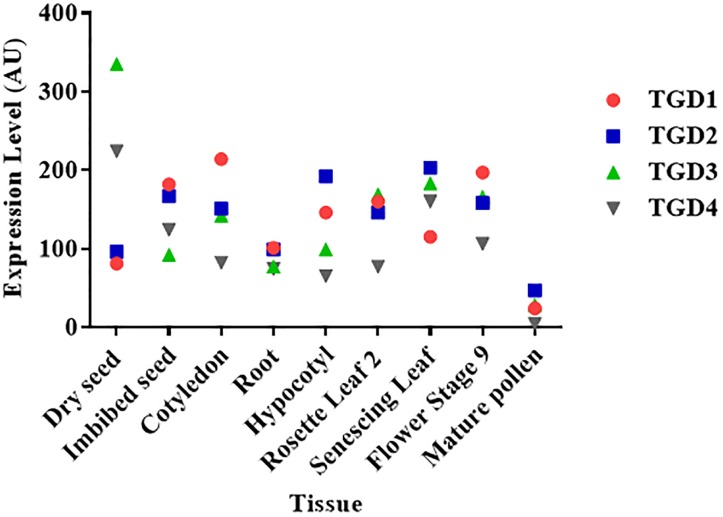
Level of expression of the TGD complex subunits in different *Arabidopsis thaliana* plant tissues. The expression levels of each gene in arbitrary units (AU) were retrieved from the Arabidopsis eFP browser (Winter et al., 2007). Data for the TGD5 subunit were not available.

According to the essential role of galactoglycerolipids in thylakoid biogenesis and photosynthesis, it is thought that lipid transfer from the ER is a crucial process that has to be tightly regulated during plant development. In accordance with this, TGD mutant plants present severe growth and developmental defects, such as defects in embryo and seed development, as well as impairments in photosynthesis, thylakoids development, and chloroplasts division (Xu et al., 2005; Fan and Xu, 2011; Li et al., 2012). According to transcriptomic data available on the Arabidopsis eFP browser (Winter et al., 2007), the TGD proteins are expressed in most of the plant tissues, supporting a role of this complex throughout plant development (Figure 3). Because of the different pathways that might be involved in plastids lipid transport (Figure 2), it would be of interest to investigate the contribution of each pathway during the different stages of plant development.

### Lipid Trafficking at ER-PM MCSs

The non-vesicular transfer of lipids from the ER to the PM in plants is suggested by pulse chase experiments showing that the transfer of several phospholipids is reduced but not blocked when vesicular trafficking is inhibited (Moreau et al., 1998). However, whether this transfer occurs at ER-PM contact sites and the actors and mechanisms involved are still unknown. These last years, several proteins located at ER-PM junctions were identified in plants (Perez-Sancho et al., 2016; Bayer et al., 2017). By homology with what is known from yeast and mammalian cells, it is thought that ER-PM contact sites in plants would also be involved in lipid transfer and that some actors might be conserved [for review, see (Perez-Sancho et al., 2016; Chang et al., 2017; Wang et al., 2017; Saheki and De Camilli, 2017a)].

In peas, an ER fraction associated to the PM, called PAM (PM associated membrane), was isolated (Larsson et al., 2007). A LPCAT activity has been detected in PAM and in the PM, indicating that PC could be exchanged at ER-PM MCSs *via* the lyso-PC intermediate, as it has been suggested at ER-plastids MCSs (see above) (Larsson et al., 2007).

Two families of ER proteins, the VAP and the E-Syt, involved in MCSs formation and lipid homeostasis in yeast and mammals, are conserved in plants (Perez-Sancho et al., 2016; Saheki and De Camilli, 2017a,b). VAP proteins interact with several LTPs, especially from the ORP family, at different MCSs (Murphy and Levine, 2016; Saheki and De Camilli, 2017a). In *A. thaliana*, three VAP homologs (VAP27-1, VAP27-3, and VAP27-4) have been located at ER-PM junctions and VAP27-3 interacts with ORP3a, a LTP able to bind and extract sitosterol *in vitro* (Saravanan et al., 2009; Wang et al., 2016). E-Syts are proteins tethering ER and PM in yeast and mammals and are able to transport lipids *in vitro* (Saheki and De Camilli, 2017b). In *A. thaliana*, AtSyt1 is a member of the E-Syt LTP family involved in the maintenance of PM integrity (Lewis and Lazarowitz, 2010) and endosome recycling (Lewis and Lazarowitz, 2010; Kim et al., 2016). AtSyt1 is located in the ER, shows specific enrichment at contact sites with PM and seems to be required for the maintenance of these junctions (Levy et al., 2015; Perez-Sancho et al., 2015; Siao et al., 2016). Despite its ability to bind *in vitro* liposomes and several phospholipids such as PA, phosphatidylinositol-phosphates or phosphatidylserine (Schapire et al., 2008; Perez-Sancho et al., 2015), the direct role of AtSyt1 in lipid transport is still elusive. It might have an indirect role by regulating ER-PM apposition. The lipid-binding activity of AtSyt1 is mediated by the two C2 domains of the protein and can be Ca^2+^ dependent, suggesting that ER-PM MCSs might be regulated by Ca^2+^ signaling (Schapire et al., 2008). In addition, AtSyt1 is involved in plant responses to different stresses, including osmotic, salt, mechanical stresses or Ca^2+^-dependent freezing tolerance, showing the importance of this protein and of ER-PM MCSs in PM homeostasis during stress (Eckardt, 2008; Schapire et al., 2008; Yamazaki et al., 2008; Perez-Sancho et al., 2015). These first examples of proteins located at ER-PM junctions in plants support the involvement of such structures in lipid homeostasis. However, further investigations are required to identify the actors and regulatory mechanisms involved, particularly in stress conditions.

Analysis of ER-PM MCSs during plant development has revealed that these structures are present in all plant tissues, including, roots, hypocotyls and leaves, but are more abundant in young tissues and elongating cells, suggesting an important role of ER-PM MCSs during active PM biogenesis (McFarlane et al., 2017). The distance between the ER and the PM is ≤15 nm and ER-PM MCSs have an average length of 160 nm. Interestingly, ER-PM MCSs are free of ribosomes but contain vesicles and microtubules, consistent with the importance of the cytoskeleton in the regulation of the localization of some ER-PM proteins, such as VAP27 (Wang et al., 2014; McFarlane et al., 2017). Overall, ER-PM MCSs seem to be important structures implicated in the regulation of PM homeostasis during both plant development and stress response.

### Lipid Trafficking at LDs-Organelle MCSs

In plants, LDs are formed either in the chloroplast to form plastoglobuli or in the ER. Little is known about the mechanistic details in plants and most of the studies were performed in tissues that accumulate and degrade oil (i.e., seeds and pollen tubes) and therefore in ER derived LDs (Pyc et al., 2017). LDs are composed of a core of neutral lipids, mainly TAGs, surrounded by a monolayer of phospholipids. Synthesis of TAGs occurs in the ER (Figure 1) and then TAGs accumulate between the two leaflets of the ER membrane. To stabilize the ER-LD junction during the maturation of the LD, SEIPIN proteins are recruited and facilitate the incorporation of additional proteins and lipids into the growing LD (Cai et al., 2015; Salo et al., 2016). During this step, CCT1 (CTP:phosphocholine cytidyltransferase 1) is recruited to the LD surface to help the coordination of the monolayer synthesis with the expansion of the LD size (Krahmer et al., 2011). It is not known whether the accumulation of TAGs in LDs relies only on their synthesis at ER-LD MCSs, or directly on LDs as in mammals (Wilfling et al., 2013; Olzmann and Carvalho, 2018), or if specific proteins are required to transfer TAGs from the ER to the LDs. Once mature, the LDs can detach from the ER by unknown process and interact with other organelles, such as peroxisomes, mitochondria, chloroplasts or vacuoles (Gao and Goodman, 2015).

Lipid droplets and TAGs play an important role at different stages of plant development including germination, flower development or pollen tube growth (Huang, 2018; Yang and Benning, 2018). During seed germination, TAGs breakdown allows the synthesis of sucrose, which serves as the main source of energy. The hydrolysis of TAGs, catalyzed by the TAG lipase SDP1 (sugar dependent 1), releases FAs that are transported in peroxisomes by the ABC transporter PXA1 and are degraded by β-oxidation to produce acetyl-CoA (Hayashi et al., 2002; Eastmond, 2006). TAG lipolysis during germination involves the direct interaction of LDs with peroxisomes, which is regulated by sucrose availability (Cui et al., 2016). This interaction facilitates the traffic of the lipase SDP1 from the peroxisome to the LD *via* the retromer complex and the formation of peroxisome extensions (Thazar-Poulot et al., 2015). PXA1 seems to regulate the extent of interactions between LDs and peroxisomes during germination (Cui et al., 2016). However, further investigation is required to understand whether or not PXA1 is directly involved in the establishment of LDs-peroxisomes MCSs for TAG breakdown during germination and to identify the other actors involved in such processes.

## Lipid Trafficking Under Stress Condition

Many stresses, such as high light, cold or nutrient stresses, are known to induce a modification of the lipid composition of cellular membranes in plants (Sandelius and Liljenberg, 1982; Jouhet et al., 2003; Zheng et al., 2016). The most common and studied situation leading to a spectacular remodeling of membrane lipids is triggered by Pi starvation.

### Lipid Remodeling During Phosphate Starvation

**FIGURE 4:**
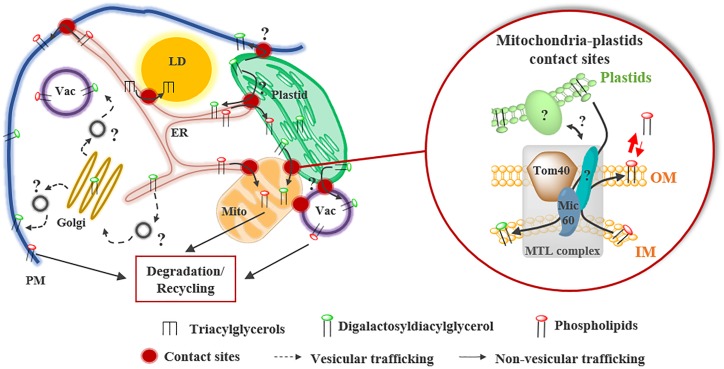
Lipid remodeling during Pi starvation in higher plants. During Pi starvation, phospholipids are partially degraded and replaced by the galactoglycerolipid DGDG synthesized in plastids. DGDG transport from plastids to mitochondria (Mito) occurs at contact sites and is partly mediated by the mitochondrial transmembrane lipoprotein (MTL) complex. Inside this complex, Mic60, located in the inner membrane (IM) of mitochondria, plays a key role in the import of DGDG from plastids and in the export of phosphatidylethanolamine from mitochondria. Mic60 interacts with Tom40 located in the outer membrane (OM) of mitochondria at OM-IM contact sites. Whether the transfer of DGDG to plasma membrane (PM) and vacuole (Vac) occurs at contact sites or via the vesicular transfer pathway is still unknown.

Phosphate starvation is a stress commonly encountered by plants that induces a wide variety of mechanisms to optimize Pi uptake from soil and Pi remobilization from intracellular reserves. As phospholipids retain up to one third of cellular Pi in plant cells, they constitute a valuable source of Pi (Poirier et al., 1991). Thus, during Pi deficiency, phospholipids are partially degraded to release Pi and are replaced by DGDG (Hartel et al., 2001; Jouhet et al., 2003; Moellering and Benning, 2011). As DGDG is synthesized in plastids, a massive transfer to mitochondria, vacuoles and the PM was shown to occur in absence of Pi (Hartel et al., 2000; Jouhet et al., 2004; Andersson et al., 2005). The transfer of DGDG from plastids to mitochondria is thought to take place at MCSs, which increase in number in this condition (Figure 4). However, whether DGDG is transported to the PM and vacuoles by vesicular or non-vesicular routes remains unknown. The transport of DGDG from plastids to mitochondria and the export of phosphatidylethanolamine for degradation or recycling are partially mediated by the Mitochondrial Transmembrane Lipoprotein (MTL) complex (Figure 4; Michaud et al., 2016, 2017). This huge complex enriched in lipids is located at MCSs between mitochondrial membranes and might also contact other organelles. AtMic60, a conserved protein of the inner membrane, plays a role in the lipid transport process (1) by regulating the proximity between mitochondrial membranes *via* its interaction with the outer membrane protein Tom40 and (2) by destabilizing membranes, likely to promote lipid desorption (Figure 4; Michaud et al., 2016). As this complex is detected in +Pi and –Pi conditions and most of the components are conserved during evolution, it might have a broader role in mitochondrial lipid trafficking.

Interestingly, analysis of the FA composition of DGDG molecules during Pi starvation has suggested that DAG backbones coming from degraded PC molecules could serve as a substrate for DGDG synthesis in plastids (Jouhet et al., 2003), raising questions about (1) the site(s) of phospholipids degradation and (2) the trafficking routes of the DAG backbones recycled from PC. Indeed, PC or PC-derivative lipids might be directly transported from mitochondria, PM and vacuoles to the plastids. Alternatively, PC molecules might be transported to the ER before being imported into plastids by the existing pathways to synthesize DGDG. The sub-cellular localization of several phospholipases, which are induced during Pi starvation and able to degrade phospholipid polar heads, suggests that phospholipids might be degraded at the surface of organelles. The vacuolar phospholipase D, PLDζ2, is localized at vacuoles-mitochondria and vacuoles-plastids MCSs during Pi starvation, suggesting that PA can be formed at the surface of mitochondria and plastids (Yamaryo et al., 2008). In addition, the phospholipase C NPC5 is localized in the cytosol and might associate with different organelle membranes to form the DAG backbones required for DGDG synthesis during Pi starvation (Gaude et al., 2008). Recently, the protein LPTD1 was shown to play an important role in the synthesis of galactoglycerolipids during Pi starvation in *A. thaliana* (Hsueh et al., 2017). LPTD1 is located in the OE of plastids and is highly induced in the absence of Pi (Hsueh et al., 2017). *Lptd1* mutant plants show an alteration of the lipid remodeling during Pi starvation, in particular, no increase of the DGDG content was observed in this mutant (Hsueh et al., 2017). Thus, LPTD1 could play a role in the recycling of phospholipid backbones in response to Pi starvation. As LPTD1 is a homolog of the TGD4 protein (Hsueh et al., 2017), it would be interesting to know whether LPTD1 is also part of the TGD complex and whether it is involved in lipid transfer from the ER and/or from other organelles to plastids to sustain phospholipids recycling in this situation.

### Symbiosis During Nitrogen Starvation

In response to some nutrient stresses, especially nitrogen starvation, a wide variety of vascular plants establish a symbiotic relationship with specific soil microorganisms to favor nutrient uptake (Harrison and Ivanov, 2017). As an example, the symbiosis between legumes and soil nitrogen-fixing bacteria called rhizobia is crucial to cope with nitrogen-deficient environments. This symbiosis leads to the formation of a specific organ, the root nodule, where nutrient exchanges occur between the host and the bacteria (Udvardi and Poole, 2013; Downie, 2014). Rhizobia invade nodule cells by endocytosis, leading to the development of symbiosomes, an intracellular compartment where rhizobia divide and eventually differentiate into nitrogen-fixing bacteroids (Udvardi and Poole, 2013). Symbiosomes are surrounded by a membrane, the PBM originating from the host cell, and can occupy a large part of the cytosol of infected cells (Udvardi and Poole, 2013; Downie, 2014). Thus, a tremendous amount of lipid synthesis and transport is required to sustain PBM synthesis and symbiosome development. The PBM seems to arise from the ER and the Golgi *via* the vesicular trafficking pathway (Whitehead and Day, 1997; Harrison and Ivanov, 2017). However, in soybean and Chinese vetch nodules, a significant amount of DGDG was detected in PBM, suggesting that plastids are also a source of lipids for PBM biogenesis (Gaude et al., 2004; Lei et al., 2014). More recently, different LTPs, AsE246 in Chinese milk vetch, and MtSyt1, MtSyt2, and MtSyt3 in *Medicago truncatula*, were shown to be involved in nodulation and development of symbiosomes (Lei et al., 2014; Gavrin et al., 2017). AsE246 is highly expressed in nodules, localized to the PBM and is able to bind several glycerolipids, such as PC and DGDG (Chou et al., 2006; Lei et al., 2014). MtSyt1, MtSyt2, and MtSyt3, which belong to the E-Syt LTP family, are highly abundant in infected nodule cells and are located to region of membrane expansion. In particular, MtSyt1 is abundant in the PBM and in the ER (Gavrin et al., 2017). These exciting results suggest that non-vesicular lipid trafficking could also be involved in the biogenesis of membranes required for the establishment of symbiosis between plant cells and microorganisms. However, further investigation is required to understand the function of LTPs and non-vesicular lipid transfer in membrane expansion during symbiosis and if lipid transfer occurs *via* MCSs between ER or plastids and PBM.

### Accumulation of Lipid Droplets During Stress

Lipid droplets have a well-known function in energy storage but proteomic analyses of plant LDs showed that they might also play alternative roles, particularly during stress (Pyc et al., 2017; Yang and Benning, 2018). TAG accumulation was observed during nitrogen or Pi deprivation in higher plants (Gaude et al., 2007; Pant et al., 2015; Shimojima et al., 2015; Mei et al., 2017), and such increases were also significantly enhanced in the presence of sugar (Yang et al., 2011). In *A. thaliana* cell cultures, TAG accumulation seems to be a frequent response to cell growth arrest mediated by nutrient stresses or chemical drugs, such as MTX (Mei et al., 2017). In nitrogen starvation or MTX treatment, TAGs are synthesized from DAG backbones generated from PC and incorporated in LDs. However, whereas MTX treatment does not affect membrane lipids, their levels are significantly decreased during nitrogen deficiency, suggesting that membrane lipids are re-routed to synthesize reserve lipids in this condition (Mei et al., 2017). Thus, TAG accumulation seems to be mediated by different pathways depending on the stress condition. Currently, few information is available concerning the actors involved in LD biogenesis in response to different stresses. As expected, enzymes involved in TAG synthesis, such as the DAG acyltransferase DGAT1, are overexpressed in response to nitrogen starvation and MTX treatment (Mei et al., 2017). I would be of interest to know if proteins involved in ER-LD contacts and LD biogenesis, like SEIPINS, are also overexpressed or if specific stress-response proteins are required. In addition, further investigation is required to understand how membrane lipids are transported to sustain LD biogenesis and what role this organelle plays, in addition to lipid storage in plants.

## Perspectives

The non-vesicular transfer of lipids at MCSs is a wide topic that has just started to be explored in plants. Plants are a particularly exciting model because of the wide variety of stresses triggering remodeling of MCSs and membrane lipid composition (Moellering and Benning, 2011; Mueller-Schuessele and Michaud, 2018). These stress-activated modifications constitute powerful tools to identify candidates and to dissect how contact sites are formed, how lipids are transferred and how these processes are regulated in cells. Most of the proteins identified in other organisms, such as the different families of LTPs (i.e., E-Syt, ORP) and VAP, are also conserved in plants. Interestingly, the number of homologs of these proteins are generally higher in plants than in yeast and mammals, and most of them are overexpressed during stress (Umate, 2011; Perez-Sancho et al., 2016; Wang et al., 2016). This highlights the complexity of non-vesicular lipid transport in plants and open important perspectives of investigation in this field for the next decades.

## Author Contributions

All authors contributed to the writing of the review. MM created the figures.

## Conflict of Interest Statement

The authors declare that the research was conducted in the absence of any commercial or financial relationships that could be construed as a potential conflict of interest.

## Abbreviations

DAG: diacylglycerol
DGD: digalactosyldiacylglycerol synthase
DGDG: digalactosyldiacylglycerol
ER: endoplasmic reticulum
E-Syt: extended-synaptotagmin
FA: fatty acid
IE: inner envelope of plastids
LD: lipid droplet
LTP: lipid transfer protein
MCS: membrane contact site
MGD: monogalactosyldiacylglycerol synthase
MGDG: monogalactosyldiacylglycerol
MTL: mitochondrial transmembrane lipoprotein complex
MTX: methotrexate
OE: outer envelope of plastids
ORP: oxysterol binding protein (OSBP) related protein
PA: phosphatidic acid
PAM: plasma membrane associated membrane
PBM: peribacteroid membrane
PC: phosphatidylcholine
Pi: phosphate
PLAM: plastid associated membrane
PM: plasma membrane
TAG: triacylglycerol
VAP: vesicle-associated membrane protein (VAMP)-associated protein
